# Ursids evolved early and continuously to be low-protein macronutrient omnivores

**DOI:** 10.1038/s41598-022-19742-z

**Published:** 2022-09-09

**Authors:** Charles T. Robbins, Amelia L. Christian, Travis G. Vineyard, Debbie Thompson, Katrina K. Knott, Troy N. Tollefson, Andrea L. Fidgett, Tryon A. Wickersham

**Affiliations:** 1 School of the Environment and School of Biological Sciences, Washington State University, Pullman, WA 99164-2812 USA; 2 Department of Rangeland, Wildlife and Fisheries Management, Texas A&M University, College Station, TX 77843 USA; 3 Cleveland Metroparks Zoo, 3900 Wildlife Way, Cleveland, OH 44109 USA; 4 Little Rock Zoo, 1 Zoo Dr, Little Rock, AR 72205 USA; 5 Missouri Department of Conservation, Ecological Health Unit, 3500 E Gans Road, Columbia, MS 65201 USA; 6 Mazuri® Exotic Animal Nutrition, Land O’Lakes, Inc., PO Box 66812, St. Louis, MO USA; 7 San Diego Zoo Wildlife Alliance, Nutrition Services, San Diego, CA 92112 USA; 8 Department of Animal Science, Texas A&M University, College Station, TX 77843 USA

**Keywords:** Ecology, Zoology

## Abstract

The eight species of bears world-wide consume a wide variety of diets. Some are specialists with extensive anatomical and physiological adaptations necessary to exploit specific foods or environments [e.g., polar bears (*Ursus maritimus*), giant pandas (*Ailuropoda melanoleuca*), and sloth bears (*Melursus ursinus*)], while the rest are generalists. Even though ursids evolved from a high-protein carnivore, we hypothesized that all have become low-protein macronutrient omnivores. While this dietary strategy has already been described for polar bears and brown bears (*Ursus arctos*), a recent study on giant pandas suggested their macronutrient selection was that of the ancestral high-protein carnivore. Consumption of diets with inappropriate macronutrient profiles has been associated with increased energy expenditure, ill health, failed reproduction, and premature death. Consequently, we conducted feeding and preference trials with giant pandas and sloth bears, a termite and ant-feeding specialist. Both giant pandas and sloth bears branched off from the ursid lineage a million or more years before polar bears and brown bears. We found that giant pandas are low-protein, high-carbohydrate omnivores, whereas sloth bears are low-protein, high-fat omnivores. The preference for low protein diets apparently occurred early in the evolution of ursids and may have been critical to their world-wide spread.

## Introduction

A recent study on giant pandas (*Ailuropoda melanoleuca*), which are bamboo specialists, concluded that much of their metabolizable energy (ME) comes from protein (i.e., 48 to 61%) and, therefore, they “required minimal evolutionary modification from their ancestral state (of carnivory or hypercarnivory) to deal with the macronutritional properties of bamboo”^1^. This conclusion suggests they did not evolve the capability to modulate liver catabolic activity necessary to conserve protein when consuming low protein diets. However, others have questioned this view of the over-riding importance of protein in ursid diet selection^2–4^, particularly in giant pandas^5^.

For example, wild polar bears (*Ursus maritimus*), as the ultimate marine ursid carnivore^6,7^, preferentially consumed fat to reduce dietary protein content to relatively low levels (i.e., 18 ± 2% ME) in comparison to that reported for giant pandas^4^. Similarly, brown bears (*Ursus arctos*), as the ultimate terrestrial ursid carnivore^8,9^, preferentially consumed either fats or soluble carbohydrates during ad libitum feeding studies to produce an equally low dietary protein content (17 ± 4% ME)^3^. Even wild brown bears with unlimited access to high protein, high energy salmon actively seek high carbohydrate berries to reduce dietary protein content to levels far below that reported for giant pandas^1,2,10^. Thus, even though both polar bears and brown bears readily consume meat, neither are carnivores or hypercarnivores in terms of dietary macronutrient proportions as suggested for giant pandas.

Ursids diverged from the carnivore lineage ~ 47.5 million years ago (Mya)^11^. Because ancestors of the giant panda diverged earlier (12 to 19.5 Mya) than the lineages that led to sloth bears and sun bears (4.4 Mya) and brown bears and polar bears (3.4 Mya)^11–13^, giant pandas may represent either an ancestral or transitional stage in ursid dietary evolution^1^. However, we hypothesized that evolving the metabolic capability to modulate existing catabolic enzymes and therefore thrive when consuming relatively low protein diets occurred early and was basic to the evolution of ursids. For the giant panda and its ancestors, this should not have been more challenging than evolving the 1) “thumb” with which to manipulate bamboo during feeding, 2) different cranial shape, jaw and associated muscles, and teeth necessary to process bamboo, and 3) ability to synthesize required nutrients (e.g., taurine, arachidonic acid, and vitamins A and B_12_) that would have occurred in the meat diet of the ancestral carnivore but do not occur in plants, including bamboo^11,14–16^.

The only other ursid that might require a high-protein macronutrient profile is the sloth bear (*Melursus ursinus*). They are specialized termite and ant consumers of the Indian subcontinent. Termites and ants provide as much as 95% of the sloth bear’s dietary energy during the winter, although carbohydrate-rich fruits can provide much of the nourishment at other times^17–20^. If giant pandas, sloth bears, brown bears, and polar bears are all low-protein macronutrient omnivores, the other four omnivorous bears [i.e., sun bears (*Helarctos malayanus*), Asiatic black bears (*U. thibetanus*), American black bears (*U. americanus*), and Andean bears (*Tremarctos ornatus*)] that often consume low protein, high carbohydrate fruits are likely also in that category^21^.

While several American and European zoos exhibit sloth bears^22,23^, they are one of the more challenging bears in human care. The sloth bear population in American zoos is not currently self-sustaining. The mean age at death of adults in American zoos is 16.7 yrs and in European zoos 18.1 ± 6.2 yrs^22,24,25^. Because a few live into their late 30s as do other bear species, lifespans of captive sloth bears are likely reduced by as much 20 yrs.

Deaths of adults are primarily due to biliary adenocarcinoma (65% of deaths in US zoos between 1905–2015; 48% of deaths in European zoos between 1960–2000) followed by various gastrointestinal diseases (15% of deaths in US zoos)^22,25^. Even in sloth bear rescue centers in India where tuberculosis is the dominant cause of death, the combination of liver, gall bladder, and kidney diseases account for 64% of the remaining deaths^26^. Neonatal mortality is high in both European zoos (67% die during the first 2 years of life) and U.S. zoos due to infectious diseases, stillbirths, and maternal neglect and trauma, all of which may be tied to inappropriate diets and resulting poor maternal health^22,25^.

Thus, we sought to test two nutritional hypotheses to identify what might be causing these problems. Without empirical evidence, others hypothesized that excess dietary fat might be responsible for the high incidence of biliary adenocarcinoma and inflammatory bowel disease^27^. This suggestion has been followed by numerous authors^22,28^ who have recommended dietary fat be kept to a minimum and, therefore, diets be high in carbohydrates. The second hypothesis, excess dietary protein, was recently proposed as causing the high incidence of hepatobiliary cancer and kidney disease in captive polar bears^4,29^. Nevertheless, in the absence of data on sloth bear nutritional requirements, the nutritional standards for the dog, cat, mink and fox have been recommended for developing captive sloth bear diets (i.e., high protein, low fat diets)^28^. Thus, we hypothesized that if dietary fats, carbohydrates, or proteins are inflaming the sloth bear’s liver, bile duct, or gastrointestinal tract by being in excess, that macronutrient should be avoided relative to its concentration in zoo diets. Similarly, if any macronutrient is deficient relative to what was necessary to maintain a healthy liver, bile duct, and gastrointestinal tract, that macronutrient should be sought and consumed in higher proportions than currently occurs.

To further test these ideas about the importance of macronutrients in ursid diet selection and evolution, we conducted feeding studies with both giant pandas and sloth bears. To differentiate between the preferred dietary macronutrient proportions and those driven simply by food availability in the wild, we chose to determine the macronutrient selection of captive giant pandas by offering unlimited amounts of bamboo and sloth bears by offering unlimited amounts of high value foods with very different protein, fat, and carbohydrate ratios. One could suggest that there are differences between the nutritional ecology of wild giant pandas in China and sloth bears in India and the captives used in this study. However, macronutrient preferences occur at the physiological level and should not differ between captive and wild states when foraging constraints do not limit food intake. This has been true in studies of brown bears, polar bears, and many other animals^2–4,30^.

## Results

### Giant pandas

Giant pandas preferred bamboo culm in all seasons except May when new shoots became available. Culm intake as a fraction of total dry matter intake ranged from 98% in March to 88% in January, 62% in October, and 49% in May. Except for May when shoots made up 51% of the dry matter intake, leaves made up the remainder (i.e., ranging from 0% in May to 2% in March, 12% in January, and 38% in October).

Protein content of culm was low in all seasons (4.2 ± 0.8% of the dry matter, ranging from 3.0% in May to 4.7% in January and March), which was approximately 25% of that in leaves (17.1 ± 2.2%, ranging from 15.2% in March to 19.6% in January) and 40% of that in new shoots in May (10.5%). While the hemicellulose content of leaves was similar throughout the seasons (31.8 ± 2.0%, ranging from 29.1% in March to 33.8% in January) and did not show a directional trend, hemicellulose content of the culm declined consistently from January (27.2%) to October (21.6%). Hemicellulose content of new shoots was 33.0% in May. Starch content of the culm declined from January (6.1%) and March (8.9%) to May (3.6%) and October (2.9%). However, the average culm starch content (5.4 ± 2.7%) was > 10 times higher than that of leaves (0.5 ± 0.3%) and shoots (0.3%). Fat was minimal in both culm (0.6 ± 0.1%) and leaves (1.9 ± 0.2%).

Digestion of bamboo hemicellulose averaged 46 ± 9% across all seasons but decreased from January (56%) to October (33%) (F_(3,3)_ = 12.020, *p* = 0.0353) and did not differ between individuals (F_(1,3)_ = 5.209, *p* = 0.1067). Starch digestibility averaged 83 ± 4% in January, March, and October when culm was preferred, but fell to 47% in May when shoots with minimal starch dominated the diet. Apparent protein digestibility averaged 48 ± 10% and did not differ across seasons (F_(3,3)_ = 0.422, *p* = 0.752) or individuals (F_(1,3)_ = 0.277, *p* = 0.666).

**Figure 1:**
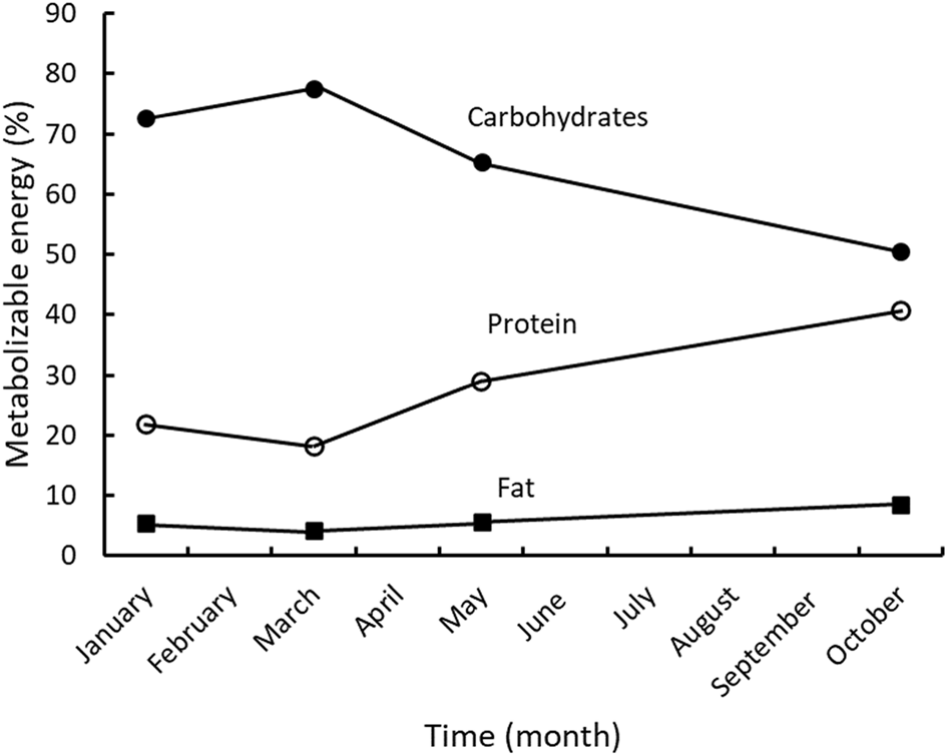
The macronutrient metabolizable energy sources determined when two adult giant pandas consumed ad libitum bamboo across seasons at the Memphis Zoo.

Carbohydrates were the dominant source of ME across all seasons and ranged from 73 and 78% in January and March to 65% and 51% in May and October (mean = 67 ± 12%, Fig. 1). Conversely, protein accounted for 22% and 18% of the ME in January and March before increasing to 29% and 41% in May and October (mean = 27 ± 10%) as dietary starch and hemicellulose content and digestibility decreased. Fat was the least important and averaged 6 ± 2% ME across seasons.

### Sloth bears

**Figure 2:**
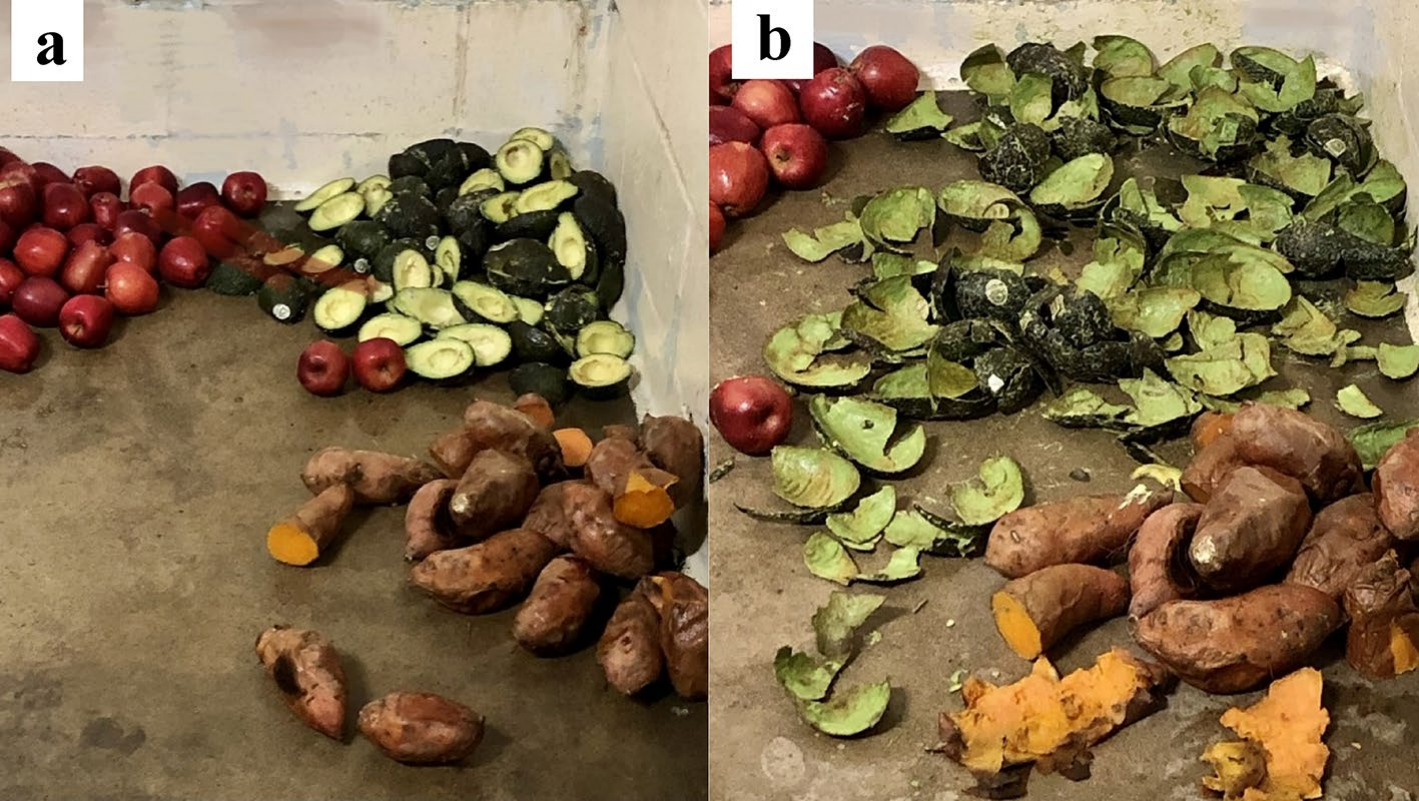
Foods offered to an adult sloth bear (**a**) and the residue left at the end of a meal (**b**). Note the clear focus on avocado flesh and minimal consumption of baked yams. No apples were consumed by any bear during the 10-day study. The whey feeder was attached elsewhere in the pen and is not shown (Photos c/o Travis Vineyard).

**Figure 3:**
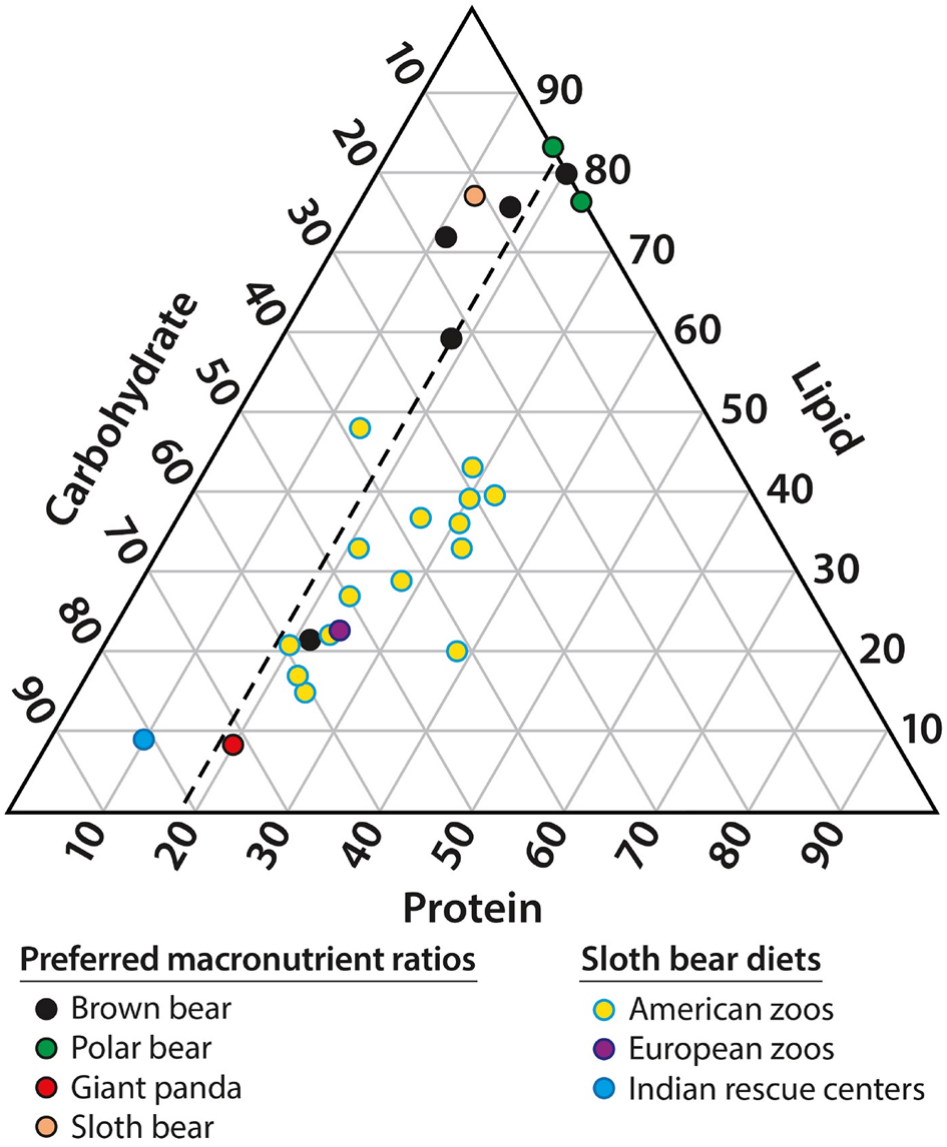
Distribution of the metabolizable energy ratios (%) selected by captive brown bears, giant pandas, sloth bears, and captive and wild polar bears. The giant panda point is for when digestible carbohydrates in bamboo were most available in the current study (i.e., January and March) and for diets fed in five Chinese zoos^32^. The dashed line is the average dietary protein concentrations selected by giant pandas, brown bears, polar bears, and sloth bears. These macronutrient preferences by ursids are in comparison to the macronutrient ratios in sloth bear diets in American zoos (e.g., Cleveland, Little Rock, Seattle, San Diego, Miami, and Philadelphia), European zoos (average for 26 zoos), and Indian rescue centers^3,4,22^. When multiple diets were submitted by American zoos, they were analyzed and are shown separately.

Even though all sloth bears consumed apples, avocados, and baked yams as part of their normal diet or during the pretrial, no apples were consumed by any bear during the 10-day ad libitum study (Supplementary Table S1 online). Thus, the dietary choices for all bears became avocados, baked yams, and whey. Avocados were strongly preferred over baked yams by all bears as avocados averaged 88 ± 15% of intake of those two items (fresh weight basis) (Fig. 2). There were no differences in the mean percentage of avocados and baked yams in the diet by day of study (F_(1,48)_ = 2.640, *p* = 0.111) nor between bears and day of study (F_(5,48)_ = 1.250, *p* = 0.301). Thus, the selected dietary ratios of avocados and baked yams did not change during the 10-day study nor did the macronutrient ME distribution. Dry matter intake averaged 36 ± 9 g/kg^0.75^/day, and its macronutrient distribution averaged 45 ± 12% fat, 14 ± 11% carbohydrate, and 14 ± 11% protein. Macronutrient ME distribution averaged 77 ± 14% fat, 11 ± 10% carbohydrate, and 12 ± 10% protein (Supplementary Table S1 online). Thus, all bears selected high fat, low carbohydrate, low protein diets (Fig. 3). Five of the 6 bears either maintained their mass or gained during the 10-day study.

## Discussion

The giant panda’s preference for culm over leaves occurred even though leaves had far more protein than did culm, which is inconsistent with the suggestion that giant pandas are high protein carnivores^1^. The giant panda’s preference for culm over leaves in the spring was likely driven by the increased availability of mono- and polysaccharides in culm relative to leaves^31^. This preference by giant pandas for a high-carbohydrate, low protein diet is similar to the brown bear’s preference for carbohydrate-rich but protein-poor berries or apples over protein- and energy-rich salmon, although both needed to be consumed to produce the most efficient diet^2,10^. The preference for culm over leaves created a protein ME in the diet of giant pandas from January to March (~ 20%) when digestible carbohydrates were most plentiful and for the entire year (27 ± 10%) that was comparable to the macronutrient proportions in giant panda milk and the milk and diets selected by other ursids (Table 1, Fig. 3) that minimize energy expenditure and maximize the efficiency of gain^3^.

**Table 1:** The protein and fat metabolizable energy concentrations (%) in ursid milks and in the diets selected by brown bears, polar bears, and sloth bears when given ad libitum access to foods rich in protein, fat, and digestible carbohydrates (PFC) or protein and fat only (PF)^1,3,4,29,32,40,54,55^.

Species	Time, Diet	Milk		Adult diet	
		Protein	Fat	Protein	Fat
Black bear, captive	Hibernation	14	82	–	–
	Post-hibernation	25	73	–	–
Brown bear, captive	Hibernation	14	82	–	–
	Post-hibernation	25	73	–	–
	Annual, PFC	–	–	17 ± 4	72 ± 9
	Fall, PF	–	–	20	80
Giant panda	–	22	70	–	–
Captive, Memphis Zoo	January to March	–	–	20	–
Captive, Chinese zoos	Annual	–	–	19 ± 4	–
Polar bear, wild	4 to 16 months	14	82	–	–
Captive	Annual, PF	–	–	24 ± 7	76 ± 7
Wild	Annual, PF	–	–	18 ± 2	82 ± 2
Sloth bear, captive	–	28	66	–	–
	Annual, PFC	–	–	12 ± 10	77 ± 14
	Average	21 ± 5	75 ± 6	18 ± 4	77 ± 2

The protein metabolizable energy values for the diets of captive giant pandas are for when they were given either ad libitum access to bamboo in the current study at the Memphis Zoo or fed bamboo and other carbohydrate-rich foods in Chinese zoos^32^.

Relative to the suggestion that giant pandas are not well adapted to consuming the more omnivorous macronutrient proportions characteristic of the diets of other ursids^1^, captive giant pandas are often fed various combinations of bamboo and high-carbohydrate supplements that include rice, baby cereal, bread, beans, wheat, millet, apples, carrots, ground corn, sorghum, sugar cane, and sugar in addition to milk, eggs, vegetables, and various meats^5,32,33^. The dry matter of giant panda diets in five Chinese zoos in which successful reproduction occurred (i.e., Beijing Zoo, Chengdu Zoo, China Conservation and Research Center, Fuzhou Zoo, and Xian Zoo) averaged 11.6 ± 2.4% protein, 39.0 ± 13.6% neutral detergent fiber (NDF) or cell wall, 5.0 ± 2.0% fat, and 5.4 ± 0.6% ash^32^. If we estimate soluble carbohydrates as 100 – (NDF + protein + fat + ash)^3^, the soluble carbohydrate content was 39.0 ± 11.2%. This approach likely underestimates digestible carbohydrates in that it assumes a zero digestibility for the hemicellulose fraction of the NDF. However, even with these assumptions, the average macronutrient ME distribution was 19 ± 4% protein, 18 ± 7% fat, and 63 ± 18% carbohydrate, or again a low-protein macronutrient ratio typical of the other ursid diets (Table 1).

Several errors may have been made in the previous giant panda study^1^ that likely influenced their conclusion. These included initially air-drying their bamboo samples in a dark room prior to laboratory drying and analyses^34^. When plants are cut and allowed to dry slowly, soluble carbohydrates are lost as they are metabolized to carbon dioxide, water, and energy until death of the plant cells^35,36^. The loss of soluble carbohydrates increases when drying occurs slowly, as would occur with air-drying in a dark room. Protein also may be metabolized, but the nitrogen remains and is only converted to different nitrogen-containing compounds, such as amides, free amino acids and peptides that would be part of a crude protein estimate^36^.

Thus, if there are significant amounts of soluble carbohydrates in fresh bamboo, air-drying of bamboo samples will lead to an underestimate of the importance of carbohydrates and thereby an overestimate of the importance of protein. Indeed, starch accounted for 16 ± 11% of the digestible macronutrients and 23 ± 13% of the digestible carbohydrates in bamboo during the current study. Also, the previous study^1^ assumed a hemicellulose digestibility of 22%^37^, which significantly underestimated that found in our digestion studies (46 ± 9%).

Another potential error in the previous study^1^ was in using a concept they termed “relative efficiencies” of macronutrient absorption in which the macronutrient profiles of bamboo were directly compared to that of giant panda feces. Such a comparison is often meaningless without knowing the amounts of food consumed and feces produced because the proportions of macronutrients in the feces reflect the extraordinarily complex interaction between the variable absorption of digestible products, passage of indigestible components, and excretion of metabolic products. Thus, only by providing data showing a close linkage between relative efficiencies and digestibility or measuring digestibility as we did can one be certain of estimating the relative importance of macronutrients.

The macronutrient intake of wild sloth bears has not been measured, although the dietary proportions and energy content of termites, ants, and fruits have been estimated^17^. Soldiers and worker termites and ants are generally low in fat and high in protein (excluding the nitrogen in their chitin exoskeleton), whereas alate and alate nymphs (winged reproductive termites) can be very low in protein and high in fat (i.e., > 50% fat)^38^. Joshi et al.^17^ surmised that sloth bears consumed primarily termite eggs and defending soldiers based on the residues in bear feces and the absence of eggs and soldiers at termite mounds after sloth bear feeding bouts. Although not measured, the dry matter of termite eggs is likely high in both protein and fat, which would create a high fat ME because of the much greater energy content of fat than protein^39^. The high fruit diet of the summer will be low in protein and fat and high in carbohydrates if not supplemented with other fat-rich foods (e.g., grubs or insect larvae)^17^. Thus, depending on season and which stage of the ant and termite life cycle the bears consume, wild sloth bears could be consuming either high or low-protein or fat diets.

The preference for fat that we observed differs markedly from current zoo diets. Zoo diets can be classified into two macronutrient types: 1) high carbohydrate, low protein, low fat diets that use grains, often in cooked porridges or soups, with fruits and vegetables or 2) diets having more modest or intermediate levels of protein, fat, and carbohydrates that include dog food, bear chows, or omnivore dry or canned products supplemented with fruits and vegetables (Fig. 3). Examples of the first type of diet are more common in Germany [e.g., Leipzig Zoo (ME protein 11%, fat 5%, and carbohydrate 84%)] and the various bear rescue centers in India [e.g., Bannerghatta Bear Rescue Centre (ME protein 10%, fat 9%, and carbohydrate 81%)]. Examples of the second type of diet are more common in US and other European zoos and have more protein and fat than the high grain diets but are much lower in fat than what bears selected in the current study^22^ (Fig. 3). Nevertheless, bears consuming all past and current zoo diets are prone to developing hepatobiliary cancer and inflammatory bowel disease.

If these problems are dietary in origin and not due to something unique to feeding on termites and ants (e.g., development of a unique gastrointestinal microbiome or consumption of formic acid in ants or chitin in both ants and termites), there are two broad types of diets not fed in captivity (i.e., high protein diets and high fat diets) (Fig. 3). In evaluating if either one of those might be more suitable for sloth bears, the protein ME ratios of ursid milks and the diets voluntarily selected by brown bears, polar bears, giant pandas, and sloth bears are low and do not differ from each other (t_(3)_ = 2.449, *p* = 0.092), which minimizes maintenance energy requirements and maximizes the efficiency of gain^1,3,4,29,40^ (Table 1). Additionally, brown bears and sloth bears prefer high fat, low carbohydrate diets when given a choice between foods rich in either carbohydrates or fats^3^ (Table 1, Fig. 3). This fat preference in the adult ursid diet is virtually identical to that occurring in ursid milks (t_(2)_ = -0.726, *p* = 0.543) even though omnivorous ursids likely have a strong preference for sweet flavors^41^.

While an understanding of the link between dietary macronutrient content and biliary cancer is lacking, we hypothesize that bears, such as polar bears and apparently sloth bears that prefer or evolved to consume high-fat diets, have high resting rates of bile production. Consequently, when sloth bears consume a high-carbohydrate, low-fat diet long term, bile is not secreted into the digestive tract as fast as it is being produced and may back up in the bile ducts, cause bile duct dilation and inflammation, and ultimately biliary cancer. An example of this process is a rare congenital disease in humans and other animals known as choledochal cyst disease. Sacs or outpocketings may develop along the bile ducts in this disease. Bile sitting in those sacs or in the bile ducts causes inflammation of the duct walls and, if not treated by surgical excision, biliary cancer^42^.

If we assume the macronutrient characteristics of ursid milks and the preferences for low protein, low carbohydrate, high fat diets exhibited by brown bears, polar bears, and sloth bears are healthy, current and past sloth bear zoo diets have provided too little fat, too much digestible carbohydrate, and often too much protein (Fig. 3). While this mismatch between the diets fed in captivity and what sloth bears prefer might explain the high incidence of hepatobiliary cancer, inflammatory bowel disease, and poor reproduction world-wide, we cannot dismiss the possibility that the bears’ preference for avocados and fat and the avoidance of apples, baked yams, and digestible carbohydrates in the current study has nothing to do with their macronutrient content and would be unhealthy long-term. Thus, additional feeding studies are needed to determine if a high fat, low protein, low carbohydrate diet might be the key to improving the health, reproduction, and longevity of captive sloth bears.

Finally, the selection of lower protein diets by giant pandas, polar bears, sloth bears, and brown bears and the often low-protein omnivorous diets of the other four ursids indicate that all ursids can modulate liver catabolic enzyme activity when needed to conserve protein. This would suggest that this ability to conserve protein occurred early in the evolution of ursids from a high protein carnivore ancestor and may have been critical to the spread of ursids world-wide by opening niches that could not be filled by another high protein carnivore. While all ursids at times may consume foods with a much higher protein content than that of a low protein omnivore, that selection process can only be evaluated relative to the other available dietary choices interacting with foraging and metabolic constraints and does not indicate their preferred diet is that of a high protein carnivore^2,43,44^.

## Methods

### Giant panda feeding study

Because plant proteins and fats are generally highly digestible when consumed by ursids whereas plant carbohydrates can range from indigestible fiber to highly digestible sugars and starch^45^, we conducted four feeding trials with a 16-yr old adult male and a 14-yr old adult female housed at the Memphis Zoo (Memphis, TN)^46^. Giant pandas were housed in separate indoor, air-conditioned enclosures during the day and moved to different enclosures at night. Access to an outdoor exhibit was offered in cooler weather. The timing of the trials coincided with the natural seasonal changes in availability and selection of differing bamboo parts. For example, two trials were timed to correspond with the period of maximum culm consumption (January 3–5, 2015; March 23–25, 2015), one trial when new shoots were consumed (May 21–23, 2015), and one trial when significant amounts of leaves were consumed (October 27–30, 2015). Although giant pandas will consume other foods^5,32,33,37^, we were interested in understanding their specialization and selection for bamboo as the seasons progressed. Thus, no other foods were fed during the trials other than small treats to move or weigh the pandas.

Bamboo was provided ad libitum, and new bamboo was offered several times per day. Bamboo was harvested locally prior to feeding, bundled by species, and stored at 16 °C under misters. Across all trials, bamboo species offered were: *Phyllostachys aureosulcata, P. bissetii, P. nuda, and Pseudosasa japonica*. All total collection digestion trials except for the one in October occurred over the course of three days, or approximately 4 × the maximum passage rate of the giant panda^47^. The October trial included an additional day.

Fresh bamboo samples (approximately 2 kg) were randomly drawn from the bamboo bundles and weighed, and the remaining bamboo in the bundle was fed to the giant pandas. Rejected bamboo culms, leaves, branches, and the culm coverings, which were pieces of the culm’s exterior layer peeled away by the giant panda as they consumed the pith, were collected. After their removal from the animal enclosure, these rejected portions were sorted and weighed. Approximately 2 kg of the whole bamboo portion and 10% of the culm coverings were randomly sampled. Bamboo offered and rejected were separated by hand into culm, culm covering, leaf, and branch fraction to estimate plant part proportions of the bamboo offered, rejected, and consumed. Samples of each fraction of bamboo and the composited feces from each animal were weighed and dried in a forced-air oven at 60 °C until reaching a constant partial dry matter weight.

### Sloth bear feeding study

We conducted six feeding trials with four males (4 yrs old, 97 kg; 5 yrs old, 140 kg; 9 yrs old, 147 kg; 15 yrs old, 117 kg) and two females (3 yrs old, 102 kg; 3 yrs old, 92 kg) between November 8, 2021, and May 22, 2022, at the Little Rock Zoo, Cleveland Metroparks Zoo, and San Diego Zoo. The selection of foods used in the sloth bear study posed a challenge as termites, ants, other invertebrates, and the fruits they consume in the wild are either not available or are prohibitively expensive. Thus, we sought foods that they would consume, were affordable, and provided very different macronutrient proportions. These foods included fat-rich avocados (*Persea americana*) and carbohydrate-rich apples (*Malus domestica*) and baked yams (*Dioscorea* sp.). We provided two carbohydrate sources and only one fat source to test the hypothesis that sloth bears would prefer a high carbohydrate diet as is currently fed world-wide (Fig. 3). Baking of yams was necessary to maximize palatability and starch digestibility^45^. While the bears had previously consumed avocados, apples, and baked yams, the selection of a protein-rich food was more challenging as they would not consume raw or cooked chicken, beef, horse meat, various fish, soybean meal, or dried and reconstituted egg whites. The final protein-rich food we tried which the bears consumed was Optimum Nutrition (Downers Grove, IL) vanilla ice cream whey powder diluted 15.5 g/100 g water (Table 2).

**Table 2:** Macronutrient composition of the foods offered to sloth bears used to determine their preference for protein, fat, and digestible carbohydrates.

Diet items	Dry matter (%)	% of dry matter			% of metabolizable energy		
		Protein	Fat	Carbohydrate	Protein	Fat	Carbohydrate
Apples	15.9	1.1	1.2	80.5	1.4	3.2	95.4
Baked yams	29.9	5.0	0.5	78.9	6.3	1.3	92.4
Avocados	26.8	7.5	54.9	6.7	5.9	89.2	4.9
Whey powder^a,b^	94.3	82.1	3.4	3.4	89.0	7.6	3.4

Nutritional data on apples, baked yams, and avocados from USDA^51^.

^a^Optimum Nutrition, Downers Grove, IL.

^b^The whey solution used in the study was made by adding 15.5 g of powder to 100 g water to create a 10.5% (w/w) protein solution.

Feedings occurred twice per day. Avocados, apples, baked yams, and the whey solution were provided in increasing amounts during a 4-day pretrial adjustment followed by ad libitum amounts during the last day of the pretrial and the 10-day trial. During both pre-trial and trial, all foods offered and rejected were weighed and the daily amount consumed determined. A meal ended when the bear stopped eating and voluntarily walked away from the food. The bears were weighed at the start and end of the 10-day trial following an overnight fast.

### Estimating nutritional value and macronutrient energy intake

Dried bamboo and giant panda fecal samples were homogenized using a Wiley Mill. A subsample of the bamboo and giant panda feces were dried at 105 ℃ for 24 h to determine 100% dry matter. Nutritional analyses for bamboo and the feces included neutral detergent fiber (NDF), acid detergent fiber (ADF), and acid detergent lignin^48^. Hemicellulose (HC) was estimated as the difference between NDF and ADF. Crude protein (N X 6.25) was determined using a carbon–nitrogen analyzer. Total starch content was determined by two-stage α-amylase:glucoamylase digestion and subsequent recording of absorbance at 510 nm^49,50^. Ether extract (fat) was determined by Soxhlet extraction. All nutritional analyses were performed in duplicate. Nutritional analyses for the giant panda study were performed by the University of Illinois (Urbana, IL USA) and Cumberland Valley Analytical Services (Hagerstown, MD USA). Because the protein, fat, energy, and digestible carbohydrate (i.e., starch and sugar) content of the human foods used in the sloth bear study are readily available in national data bases^51^ or from the manufacturer, we used those values to estimate the nutritional composition of the selected diets.

Digestible protein, fat, and carbohydrates (i.e., starch and hemicellulose) were measured directly in the giant panda study. Contrary to the earlier study^1^ that assumed a cellulose digestibility of 8%, we took a more conservative approach and assumed no cellulose digestion in this monogastric with a rapid rate of passage^37^. For the sloth bear studies, digestible carbohydrates were defined as starch and sugars, which are the primary digestible carbohydrates in baked yams, apples, and avocados^51^. We then summed the amounts of proteins, fats, and digestible carbohydrates consumed in each food (sloth bears) or plant part (giant pandas) to estimate their total daily intake.

To estimate metabolizable energy intake, total daily intake of each macronutrient was multiplied in the sloth bear study by 4.3 kcal/g for protein, 8.9 for fat, and 4.0 for digestible carbohydrates^52^. Because there are no specific metabolizable energy values for bamboo, we used the average for “vegetables”, which were 3.7 kcal/g for protein, 8.4 for fats, and 4.0 for carbohydrates^52^. The lower protein and fat energy equivalents for bamboo occur because plant leaves and stems have significant amounts of non-protein nitrogen, non-digestible fiber-bound nitrogen, and non-polar extractable compounds that are not triglycerides^53^. However, use of the lower ME equivalents reduced the protein ME estimate by 2.8 ± 0.6 percentage points in comparison to the higher values used in the sloth bear study and, therefore, do not affect our determination of whether giant pandas are low protein omnivores or high protein carnivores. Metabolizable energy values for ursid milks were 4.27 kcal/g for protein, 8.79 for fat, and 3.87 for carbohydrates^52^.

Methods for all aspects of data collection from both giant pandas and sloth bears were carried out in accordance with relevant guidelines and regulations and approved as specified in the above sections by the Institutional Animal Care and Use Committees of the participating zoos (Memphis Zoo, Little Rock Zoo, Cleveland Metroparks Zoo, and San Diego Zoo) and Texas A&M University. In addition, all aspects of the study are reported in accordance with ARRIVE guidelines (https://arriveguidelines.org).

### Statistical analyses

Least squares means (LSM) and standard deviations for apparent digestibilities of hemicellulose, starch, and crude protein in the giant panda study were determined using SAS Mixed Procedure, with animal as a replicate within trial (SAS 9.3, SAS Institute, Cary, NC). Two-way ANOVAs were used to test for differences in digestive efficiency of giant pandas between individuals and seasons and for interactions in diet selection between individual sloth bears and days during the study. A paired t-test was used to test for differences in protein and fat ME concentrations between ursid milks and the diets voluntarily selected in ad libitum feeding studies. Significant changes in digestibility or diet were determined at the 0.05 level. All means are reported ± 1SD.

## Supplementary Information

Supplementary Information.

## Data Availability

Data used in this study are available in the cited thesis of A.L.C.^46^ (giant pandas) and from C.T.R. and Supplementary Table S1 online (sloth bears).
