# Supplementary material for: Ursids evolved early and continuously to be low-protein macronutrient omnivores
Source: Sci Rep. 2022 Sep 9;12:15251. doi: 10.1038/s41598-022-19742-z (PMC9463165; doi:10.1038/s41598-022-19742-z)
Supplement: Supplementary file 1 — Supplementary Information. [file 41598_2022_19742_MOESM1_ESM.docx]

Supplementary Table S1. Intake values for the sloth bears used in the preference study.

______________________________________________________________________________________________________________________

Daily intake (fresh, g/day) __ ___Dry matter intake (%) Metabolizable energy intake (%)

Bear Sex Apples Yams Avocados Whey solution Protein Fat Carbohydrate Protein Fat Carbohydrate

Shala F 0 0 4431 0 7.5 54.9 6.7 5.9 89.2 4.9

Zaara F 0 520 1492 2300 34.4 28.4 19.6 29.0 58.2 12.8

Bala M 0 285 6686 6 7.5 54.6 7.0 5.9 89.0 5.1

Kartick M 0 24 4446 90 8.4 54.1 7.1 6.6 88.2 5.2

Pabu M 0 212 3835 1845 22.0 43.2 9.6 18.3 74.3 7.4

Sahaasa M 0 1575 2677 0 6.5 33.3 35.3 6.0 63.7 30.3

______________________________________________________________________________________________________________________
